# Robust Multi-Objective Optimization for Response Surface Models Applied to Direct Low-Value Natural Gas Conversion Processes

**DOI:** 10.3390/e23020248

**Published:** 2021-02-21

**Authors:** Luiz Célio S. Rocha, Mariana S. Rocha, Paulo Rotella Junior, Giancarlo Aquila, Rogério S. Peruchi, Karel Janda, Rômulo O. Azevêdo

**Affiliations:** 1 Department of Management, Federal Institute of Education, Science and Technology—North of Minas Gerais, Almenara 39900-000, Brazil; luizrochamg@hotmail.com; 2 Faculty of Pharmacy, Fluminense Federal University, Niterói 24241-000, Brazil; mariana.souzarocha@yahoo.com.br; 3 Department of Production Engineering, Federal University of Paraiba, João Pessoa 58051-900, Brazil; rsp@academico.ufpb.br (R.S.P.); romulocpm@hotmail.com (R.O.A.); 4 Faculty of Finance and Accounting, Prague University of Economics and Business, 13067 Prague, Czech Republic; karel-janda@seznam.cz; 5 Institute of Production and Management Engineering, Federal University of Itajuba, Itajuba 37500-903, Brazil; giancarlo.aquila@yahoo.com

**Keywords:** low-value natural gas, carbon dioxide oxidative coupling of methane, robust multi-objective optimization, normal boundary intersection, entropic measure

## Abstract

The high proportion of CO_2_/CH_4_ in low aggregated value natural gas compositions can be used strategically and intelligently to produce more hydrocarbons through oxidative methane coupling (OCM). The main goal of this study was to optimize direct low-value natural gas conversion via CO_2_-OCM on metal oxide catalysts using robust multi-objective optimization based on an entropic measure to choose the most preferred Pareto optimal point as the problem’s final solution. The responses of CH_4_ conversion, C_2_ selectivity, and C_2_ yield are modeled using the response surface methodology. In this methodology, decision variables, e.g., the CO_2_/CH_4_ ratio, reactor temperature, wt.% CaO and wt.% MnO in ceria catalyst, are all employed. The Pareto optimal solution was obtained via the following combination of process parameters: CO_2_/CH_4_ ratio = 2.50, reactor temperature = 1179.5 K, wt.% CaO in ceria catalyst = 17.2%, wt.% MnO in ceria catalyst = 6.0%. By using the optimal weighting strategy w_1_ = 0.2602, w_2_ = 0.3203, w_3_ = 0.4295, the simultaneous optimal values for the objective functions were: CH_4_ conversion = 8.806%, C_2_ selectivity = 51.468%, C_2_ yield = 3.275%. Finally, an entropic measure used as a decision-making criterion was found to be useful in mapping the regions of minimal variation among the Pareto optimal responses and the results obtained, and this demonstrates that the optimization weights exert influence on the forecast variation of the obtained response.

## 1. Introduction

A mixture of various hydrocarbons such as methane (CH_4_), ethane (C_2_H_6_), propane (C_3_H_8_), butane (C_4_H_10_) and inert diluents such as molecular nitrogen (N_2_) and carbon dioxide (CO_2_) are found in natural gas compositions. Natural gas composition variations are affected by several parameters such as the geographical source, the time of year, and the treatment applied during production or transportation [1,2,3].

CH_4_ and CO_2_ are believed to contribute to the greenhouse effect [4]. Although the amount of CH_4_ in the atmosphere is less than CO_2_, its global warming potential is approximately 25 times greater [5]. Large amounts of methane are widely available in nature in the form of natural gas, although methane is a greatly underutilized resource for chemical and liquid fuels [6].

Large amounts of natural gas reserves are located in remote areas [7,8] and pipelines may not be available to transport that remote gas to regions where it can be used, and liquefication for transportation via ship is quite expensive. Almost 11% of this gas is reinjected, while another 4% is flared or vented [5,9,10], generating waste [10].

The conversion of CH_4_ and CO_2_ into value-added chemicals has attracted attention from the academic community and industries [4,11,12,13]. The abundance of these two gases makes them stand out as raw materials for fuels and chemical synthesis. Furthermore, CH_4_ is considered to be the cheapest carbon source for the petrochemical industry [14].

Among these reserves, there are large amounts of low-value natural gas containing a high concentration of CO_2_. Currently this natural gas is sold as liquefied natural gas after a carbon dioxide clean up using CO_2_ separation facilities. The impurities from both the separation stages are then injected underground to dispose of the waste using a sub-surface aquifer, but at an estimated 49% of the total cost of the project [15]. The high CO_2_/CH_4_ ratio in low-value natural gas compositions could be strategically utilized to produce value-added chemicals, such as higher hydrocarbons and liquid fuels without having to separate the CO_2_ first [16]. The synthesis of liquid fuels and commodity chemicals from CO_2_ is a promising approach for clean energy production. For this reason, much academic and industrial effort has been devoted to exploring efficient means of reducing CO_2_ [17].

Several studies have been undertaken with the objective of making conversion of methane viable, either by direct or indirect routes. The indirect routes focus on steam reform to produce synthesis gas (CO + H_2_), which can be converted into the liquid fuels [18]. The direct route, on the other hand, converts methane into higher hydrocarbons in one step through oxidative methane coupling reactions (OCM). Therefore, it is considered more economically viable and, consequently, it has been the subject of several studies [4,12,14,16,19,20,21,22,23,24,25,26,27,28,29,30,31]. The general reactions for the formation of C_2_ hydrocarbons from methane and carbon dioxide are expressed by [24]:

(1) $$2CH_{4}+CO_{2}⇌C_{2}H_{6}+CO+H_{2}O$$

(2) $$2CH_{4}+2CO_{2}⇌C_{2}H_{4}+2CO+2H_{2}O$$

Although the OCM reaction is a highly exothermic reaction, it requires high temperatures and a suitable catalyst because the binding energy of hydrogen-carbon in methane is very large [32] and the bond dissociation energy of CO-O is also high. Because of the low reactivity of CO_2_, the product (C_2_H_4_, C_2_H_6_) of the OCM reaction, with CO_2_ as an oxidant, is less likely to react with CO_2_. Therefore, high C_2_ (C_2_H_4_, C_2_H_6_) selectivity is expected in the CO_2_-OCM [31].

Nevertheless, a possible increase in temperature will result in total oxidation instead of partial oxidation of C_2_ hydrocarbons, such as ethylene [6]. We therefore see the importance of catalyst selection for the OCM reaction. The catalyst determines at what temperature and composition the maximum yield and selectivity for the reaction will be defined [30].

Considerable efforts have been placed on developing OCM catalysts in order to make the product yields commercially feasible [4,12,14,19,20,21,23,24,27,29,30,31].

Of the various catalysts for CO_2_ activation, ceria (CeO_2_) is attracting increased interest due to its high oxygen storage capacity [11]. Indeed, oxidation and reduction reactions of Ce^4+^ and Ce^3+^ are effective in activating carbon dioxide to form oxygen active species, while the C_2_ selectivity is related to the basicity of a catalyst due to enhanced CO_2_ chemisorption on the catalyst surface [11,27]. Wang et al. [25] and Wang et al. [26] proposed using a ceria catalyst modified with CaO. According to these authors the CaO/CeO_2_ is the most effective catalyst for conversion at high temperatures of CH_4_ to C_2_H_6_ and C_2_H_4_ by CO_2_ among a series of CeO_2_ catalysts modified with alkali and alkaline earth metal oxides. Istadi and Amin [28] and Istadi and Amin [16] proposed using CaO/CeO_2_ and MnO/CeO_2_ catalysts in the multi-objective optimization of CO_2_-OCM process. According to these authors, the optimal operating parameters, such as the CO_2_/CH_4_ ratio and reactor temperature, and the catalyst compositions in the ceria catalyst, provide essential information for industrial CO_2_-OCM processes.

At the same time, multi-objective optimization allows one to find the point that represents the final solution to the problem, in addition to treating the reliability of that solution as an important factor. It then follows that the variance of the prediction would be of great concern [33].

The aim of this study is to optimize the conversion of natural gas, rich in carbon dioxide, using robust multi-criteria decision-making processes, based on an entropic measure, to determine the ideal Pareto point as the final solution to the problem. The normal boundary intersection method (NBI) together with the mixture design of experiments (MDE), will be used to optimize these responses simultaneously.

## 2. Multi-Objective Optimization 

Industrial processes, when translated into optimization problems, are usually treated as multi-objective problems, since they involve more than one desirable resource. When the objective functions are not in conflict, each objective function reaches its optimal value, and a solution is found, without the need for any special method [34]. These objectives are often conflicting [35]. A multi-objective optimization (MOP) problem can be formulated in order to study the tradeoffs between these conflicting objectives, as in:

(3) $$\underset{x\in \Omega }{Min}\;\Lambda =\left\{F_{1}\left(x\right),F_{2}\left(x\right),\ldots ,F_{k}\left(x\right)\right\}$$

where Λ is the vector of the objective functions that comprise the conflicting *k* criteria and *F_i_* is the vector of the decision variables *x* belonging to the viable set Ω. 

Their restrictions are represented in the form of inequalities or equalities, according to:

(4) $$\Omega =\{x\in {\mathbb{R}}^{n}|g_{r}(x)\leq 0,\;r\in I,\;h_{q}(x)=0,\;q\in J\}$$

where *g_r_* and *h_q_* are the inequality and equality constraint functions, respectively and *I* and *J* are the index sets containing as many elements as there are inequality and equality constraints, respectively.

When looking for an MOP solution, the goal is to find efficient solutions. Some variations in the concept of efficiency are concepts of local efficiency, weak efficiency, and weakly local efficiency. Hence, a solution $x^{*}\in X$ is locally efficient if δ>0 such that $x^{*}$ is efficient in $X\cap N(x^{*},\delta )$, where $N(x,\delta )=\{y|y\in {\mathbb{R}}^{n},\left\|x-y\right\|<\delta \}$. A solution $x^{*}\in X$ is weakly efficient if there is no other point x∈X such that $f(x)<f(x^{*})$. A solution $x^{*}\in X$ is locally weakly efficient if δ>0 such that $x^{*}$ is weakly efficient in $X\cap N(x^{*},\delta )$.

However, for a given decision vector $x^{*}$ to be the solution to an optimization problem, it must satisfy some conditions, which we call optimality conditions. The simplest situation for which we can define the optimality conditions is that in which we have a single function for which we wish to find the optimal point, and there are no constraints. In the case of unconstrained optimization, the necessary first-order condition is: 

If $x^{*}$ is to be a local minimizer of the function *f*(**x**), differentiable at $x^{*}$, then $\nabla f(x^{*})=0$.

At this point, Miettinen [34] is used, which shows that a function $f_{i}:{\mathbb{R}}^{n}\to \mathbb{R}$ is differentiable at $x^{*}$ if:

(5) $$f_{i}(x^{*}+d)-f_{i}(x^{*})=\nabla f_{i}{\left(x^{*}\right)}^{T}d+\left\|d\right\|\varepsilon (x^{*},d)$$

where $\nabla f_{i}(x^{*})$ is the gradient of $f_{i}\;\text{at}\;x^{*}$ and $\varepsilon (x^{*},d)\to 0$ as ‖d‖→0.

Furthermore, $f_{i}$ is continuously differentiable at $x^{*}$ if all of its partial derivatives $\frac{\partial f_{i}(x^{*})}{\partial x_{j}}$(j=1,…,n), i.e., all the gradient components, are continuous at $x^{*}$. Still, with respect to the unconstrained optimization, the necessary second-order condition is: 

If $x^{*}$ is to be a local minimizer of the function *f*(**x**), twice differentiable at $x^{*}$, then $\nabla f(x^{*})=0$ and $\nabla^{2}f(x^{*})$ must be positive semidefinite, i.e., it has eigenvalues ($\lambda_{i}$) greater than or equal to zero.

According to Miettinen [34], a function $f_{i}:{\mathbb{R}}^{n}\to \mathbb{R}$ is twice differentiable at $x^{*}$ if:

(6) $$f_{i}(x^{*}+d)-f_{i}(x^{*})=\nabla f_{i}{\left(x^{*}\right)}^{T}d+\frac{1}{2}d^{T}\nabla^{2}f_{i}(x^{*})d+{\left\|d\right\|}^{2}\varepsilon (x^{*},d)$$

where: $\nabla f_{i}(x^{*})$ is the gradient, the symmetric matrix, n×n; $\nabla^{2}f_{i}(x^{*})$ is a Hessian matrix of $f_{i}\;\text{at}\;x^{*}$; and $\varepsilon (x^{*},d)\to 0$ as ‖d‖→0. 

The Hessian matrix of a twice differentiable function consists of second-order partial derivatives $\frac{\partial^{2}f_{i}(x^{*})}{\partial x_{j}\partial x_{l}}(j,\;l=1,\ldots ,n)$, and it can be presented as:

(7) $$\nabla^{2}f_{i}(x^{*})=\left(\begin{array}{ccc} \frac{\partial^{2}f_{i}(x^{*})}{\partial x_{1}^{2}} & \cdots & \frac{\partial^{2}f_{i}(x^{*})}{\partial x_{1}\partial x_{n}}\\ \vdots & \ddots & \vdots \\ \frac{\partial^{2}f_{i}(x^{*})}{\partial x_{n}\partial x_{1}} & \cdots & \frac{\partial^{2}f_{i}(x^{*})}{\partial x_{n}^{2}} \end{array}\right)$$

Furthermore, $f_{i}$ is twice continuously differentiable at $x^{*}$ if all of its second-order partial derivatives are continuous at $x^{*}$.

With respect to optimality conditions in unconstrained optimization, we see that only the presented conditions are necessary, since the first and second order terms can be null, still leaving doubt about the nature of $x^{*}$. Therefore, a sufficient condition for $x^{*}$ to be a strict local minimizer of the function *f*(**x**), twice differentiable at $x^{*}$, is $\nabla f(x^{*})=0$ and $\nabla^{2}f(x^{*})$ as a positive definite, i.e., it has eigenvalues ($\lambda_{i}$) greater than zero.

To analyze the critical points the second order derivatives of the function must exist and be different from zero, which can be verified by performing a Taylor series expansion around the optimal point. Since the first derivative is null, we have:

(8) $$f_{i}(x)-f_{i}(x^{*})=\frac{1}{2}{\left(x-x^{*}\right)}^{T}\nabla^{2}f_{i}(x^{*})(x-x^{*})$$

The values of the function near the critical point depend on the Hessian. In summary, we have the following relation between the Hessian matrix eigenvalue signals and the critical point:

- if all eigenvalues are positive, we have a minimum point;
- if all eigenvalues are negative, we have a maximum point; and
- if the eigenvalues show different signs, we have a saddle point.

This analysis still allows us to deduce the convexity of the function. Similarly, when working with Response Surface Methodology (RSM), the determination of the convexity of a function is done by characterizing the nature of the stationary point. The stationary point is the level of *x*_1_, *x*_2_, …, *x_k_*, that optimizes the predicted response. This point, if it exists, will be the set of *x*_1_, *x*_2_, …, *x_k_*, for which the partial derivatives are equal to zero. A general mathematical solution for locating the stationary point may be obtained. The second-order model is expressed in the matrix notation as [36]:

(9) $$\hat{y}={\hat{\beta }}_{0}+x^{T}b+x^{T}Bx$$

where:

(10) $$x=\left[\begin{array}{l} x_{1}\\ x_{2}\\ \;\vdots \\ x_{k} \end{array}\right],\;b=\left[\begin{array}{l} {\hat{\beta }}_{1}\\ {\hat{\beta }}_{2}\\ \;\vdots \\ {\hat{\beta }}_{k} \end{array}\right],\;\mathrm{and}\;B=\left[\begin{array}{cccc} {\hat{\beta }}_{11} & {\hat{\beta }}_{12}/2 & \cdots & {\hat{\beta }}_{1k}/2\\ {\hat{\beta }}_{21}/2 & {\hat{\beta }}_{22} & \cdots & {\hat{\beta }}_{2k}/2\\ \vdots & \vdots & \ddots & \vdots \\ {\hat{\beta }}_{k1}/2 & {\hat{\beta }}_{k2}/2 & \cdots & {\hat{\beta }}_{kk} \end{array}\right]\;\mathrm{for}\;{\hat{\beta }}_{ij}={\hat{\beta }}_{ji}$$

The derivative of $\hat{y}$ with respect to the elements of the vector **x** equated to zero is:

(11) $$\frac{\partial \hat{y}}{\partial x}=b+2Bx=0$$

The stationary point described in Equation (12) is the solution of Equation (11):

(12) $$x_{s}=-\frac{1}{2}B^{-1}b$$

The predicted response at the stationary point is given by:

(13) $${\hat{y}}_{s}={\hat{\beta }}_{0}+\frac{1}{2}x_{s}{}^{T}b$$

In general terms, the nature of a stationary point can be determined from the sign of the eigenvalues or root characteristics of a given matrix **B**. The eigenvalues ($\lambda_{i}$) of the matrix **B** are the solutions to the following equation:

(14) |B−λI|=0

Then, these are defined as follows:

- if the values of $\lambda_{i}$ are all negative, the function is concave and **x**_S_ is a maximum point;
- if the values of $\lambda_{i}$ are all positive, the function is convex, and **x**_S_ is a minimum point;
- if the values of $\lambda_{i}$ present different signs, the function is neither concave nor convex, and **x**_S_ is a saddle point.

In multi-objective optimization two approaches can be used to aid in solving problems: (i) converting all objective functions into a single problem; (ii) optimizing one objective, considering other objectives as constraints [37]. In this second approach, the objective function is prioritized and the relevance of the others is relegated to a lower extent.

According to Shahraki and Noorossana [37], the methods that result in obtaining a set of optimal Pareto solutions are recommended, since they provide the best solutions among all other given options in terms of efficiency. The weighted sum method (WSM) is widely used to obtain optimal Pareto solutions in MOP, as it is easy to implement and interpret. However, if the set of Pareto optimal solutions is nonconvex, the frontier becomes nonconvex and discontinuous, forming clusters of solutions in regions of great curvature, yet discontinuous in the solution space. In such situations, the WSM, which is the standard method for generating the Pareto set in MOP, barely finds solutions in the nonconvex section. Moreover, the WSM cannot generate an equally spaced frontier, even if the distribution of weights is uniform [38,39], which can confuse the decision maker by not clarifying the conflicting behavior and the trade-offs between different objective functions.

Das and Dennis [40] presented the Normal Border Intersection (NBI) method as an option that can overcome the disadvantages presented by the WSM method. This method presents the Pareto surface distributed evenly independent of relative scales and the convexity of the objective functions.

However, Das and Dennis [40] argue that a disadvantage, inherent to methods that seek to find a large number of efficient points in MOP, is that these methods cannot find globally Pareto optimal points. The points generated by the NBI are only locally guaranteed as Pareto optimal points. Nevertheless, we will use the NBI method in this study, given its robustness when working with nonconvex problems.

Thus, the NBI method is used to solve the MOP using the following equation [40]:

(15) $$\begin{array}{ll} \underset{\left(x,\;D\right)}{Max} & D\\ s.t.: & \bar{\Phi }w-D\bar{\Phi }e=\bar{F}(x)\\ & x\in \Omega \end{array}$$

where *w* is the convex weighting; *D* is the distance between the Utopia line and the Pareto frontier; $\bar{F}(x)$ is the vector containing the individual values of the normalized objectives in each run; e is a column vector of one and Φ and $\bar{\Phi }$ are the payoff and normalized payoff matrices, respectively, and can be written as:

(16) $$\Phi =\left[\begin{array}{ccc} f_{1}^{*}\left(x_{1}^{*}\right) & \cdots & f_{1}^{}\left(x_{m}^{*}\right)\\ \vdots & \ddots & \vdots \\ f_{m}^{}\left(x_{1}^{*}\right) & \cdots & f_{m}^{*}\left(x_{m}^{*}\right) \end{array}\right]\Rightarrow \bar{\Phi }=\left[\begin{array}{ccc} \frac{f_{1}^{*}\left(x_{1}^{*}\right)-f_{1}^{*}\left(x_{1}^{*}\right)}{f_{1}^{}\left(x_{m}^{*}\right)-f_{1}^{*}\left(x_{1}^{*}\right)} & \cdots & \frac{f_{1}\left(x_{m}^{*}\right)-f_{1}^{*}\left(x_{1}^{*}\right)}{f_{1}^{}\left(x_{m}^{*}\right)-f_{1}^{*}\left(x_{1}^{*}\right)}\\ \vdots & \ddots & \vdots \\ \frac{f_{m}\left(x_{1}^{*}\right)-f_{m}^{*}\left(x_{m}^{*}\right)}{f_{m}^{}\left(x_{1}^{*}\right)-f_{m}^{*}\left(x_{m}^{*}\right)} & \cdots & \frac{f_{m}^{*}\left(x_{m}^{*}\right)-f_{m}^{*}\left(x_{m}^{*}\right)}{f_{m}^{}\left(x_{1}^{*}\right)-f_{m}^{*}\left(x_{m}^{*}\right)} \end{array}\right]$$

## 3. Criteria for Defining the Ideal Pareto Solution 

When solving an MOP, there are usually an infinite number of efficient solutions that form the ideal set of Pareto (called efficient set) points [34]. The process that seeks to generate optimal Pareto alternatives is known as multi-objective optimization. From a mathematical standpoint, every Pareto ideal point is an acceptable solution for a MOP, if the objective is to obtain a point as the final solution [41].

It is difficult to define the degree of importance to be attributed to each objective [42]. The definition of the weights for each function can be influenced by the preferences of the decision maker. This affects the influence of weights used to determine the relative importance of the functions, in order to identify the most important parameters during the optimization process, and the preferences are selected [43].

The priority given to the criteria significantly affects the final result, since this result depends on the importance attached to each objective [44,45,46]. This can become a problem as decision makers are often unsure about the exact weights of objective functions [44]. Considerable cognitive effort is necessary [47] in order to obtain information on direct preference from the analyst, 

The Pareto set includes rational options, from which the analyst can select the final solution by comparing several objectives with each other [44]. As such, the search is for a set of ideal solutions in the broadest sense (Pareto optimal). Several techniques from literature address optimal Pareto solutions in the solution space. However, the disadvantage of these methods is the variety of solutions from which one must choose. We therefore can identify a need to bridge the gap between exclusive solutions and Pareto ideal sets [44].

When solving a linear multi-objective optimization problem, Zeleny [48,49] sought to answer the following questions: (i) what is the most preferred solution among the generated, non-dominated and extreme solutions? (ii) Can the set of non-dominated solutions be reduced to consist of fewer points to determine a final decision? To answer these questions, he proposed the “traditional measure of entropy” as a parameter to assess the importance of functions and define the weights to be used in solving the problem.

Following a different approach, some authors (see [50,51,52]) used the Shannon entropy index [53] associated with an error measure, to determine the most preferred Pareto ideal point in a MOP in a vertical turning process, resolved using the NBI method. The authors state that Shannon’s entropy index can provide a more reliable assessment of the relative weights of objectives in the absence of analyst preferences. Furthermore, the authors state that when combined with an error measure, it minimized the error of Pareto’s preferred point related to individual optimal responses. The weighting metric *ξ* is obtained by [50,52]:

(17) $$\begin{array}{ll} Max & \xi =\frac{Entropy}{GPE}\\ s.t.: & \sum_{i=1}^{n}w_{i}=1\\ & 0\leq w_{i}\leq 1 \end{array}$$

where *w_i_* are the weights to be assigned to the objectives that are to be optimized.

The Entropy equation can be calculated by [53]:

(18) $$Entropy=-\sum_{i=1}^{m}w_{i}\ln w_{i}$$

The GPE equation is represented by [54]:

(19) $$GPE=\sum_{i=1}^{m}\left|\frac{y_{i}^{*}}{T_{i}}-1\right|$$

where $y_{i}^{*}$ is the value of the Pareto-optimal responses; $T_{i}$ is equal to the defined target and *m* is equal to the number of objectives.

As discussed earlier, many of the weighting strategies used during the optimization and decision-making process rely on inaccurate and subjective elements in at least one of the stages. Thus, weighting method analysis shows that significant contributions can be made, since a large portion of these strategies still employ elements susceptible to error.

Only Shahraki and Noorossana [34] proposed a way of evaluating any variability parameter when selecting the best Pareto optimal solution. We therefore see the theoretical gap, that we intend to address in this study: the behavior of the prediction variance in relation to the weighting strategies.

Now, consider the following problem [55]:

(20) $$\begin{array}{ll} \underset{x}{Min} & \sum_{i=1}^{n}w_{i}f_{i}(x)\\ s.t.: & \sum_{i=1}^{n}w_{i}=1\\ & \;\;\;\;\;\;\;\;w_{i}\geq 0,\;i=1,\ldots ,n \end{array}$$

where *f_i_*(*x*) are the objective functions to be optimized and *w_i_* are the weights assigned to each objective function.

In order to calculate the variance for the function described in Equation (20), the following process is considered:

(21) $$\begin{array}{ll} \mathrm{Var}\left[\sum_{i=1}^{n}w_{i}f_{i}(x)\right] & ={\sum_{i=1}^{n}\left[\frac{\partial w_{i}f_{i}(x)}{\partial f_{i}(x)}\right]}^{2}\sigma_{f_{i}}^{2}+2\sum_{i}^{n}\sum_{j}^{n}\left[\frac{\partial w_{i}f_{i}(x)}{\partial f_{i}(x)}\right]\left[\frac{\partial w_{j}f_{j}(x)}{\partial f_{j}(x)}\right]\sigma_{f_{i}f_{j}}\\ & =\sum_{i=1}^{n}w_{i}^{2}\sigma_{f_{i}(x)}^{2}+2\sum_{i}^{n}\sum_{j}^{n}w_{i}w_{j}\sigma_{f_{i}f_{j}}\\ & =\sum_{i=1}^{n}w_{i}^{2}\mathrm{Var}\left[f_{i}(x)\right]+2\sum_{i}^{n}\sum_{\neq j}^{n}w_{i}w_{j}\rho_{f_{i}f_{j}}\sqrt{\mathrm{Var}\left[f_{i}(x)\right]\times \mathrm{Var}\left[f_{j}(x)\right]} \end{array}$$

where $\rho_{f_{i}f_{j}}$ is the correlation between the functions *f_i_* and *f_j_*.

Considering that it is possible to calculate the variance of *f_i_* (*x*) at a certain point $x_{0}{}^{T}=\left[\begin{array}{ccccc} 1 & x_{01} & x_{02} & \ldots & x_{0k} \end{array}\right]$, as $\mathrm{Var}[f_{i}(x_{0})]={\hat{\sigma }}_{f_{i}}^{2}x_{0}{}^{T}{\left(X^{T}X\right)}^{-1}x_{0}$, Equation (21) can be modified to:

(22) $$\begin{array}{l} Var\left[\sum_{i=1}^{n}w_{i}f_{i}(x_{0})\right]=\sum_{i=1}^{n}w_{i}^{2}\left[{\hat{\sigma }}_{f_{i}}^{2}x_{0}{}^{T}{\left(X^{T}X\right)}^{-1}x_{0}\right]+\\ \;2\sum_{i}^{n}\sum_{\neq j}^{n}w_{i}w_{j}\rho_{f_{i}f_{j}}\sqrt{\left[{\hat{\sigma }}_{f_{i}}^{2}x_{0}{}^{T}{\left(X^{T}X\right)}^{-1}x_{0}\right]\times \left[{\hat{\sigma }}_{f_{j}}^{2}x_{0}{}^{T}{\left(X^{T}X\right)}^{-1}x_{0}\right]} \end{array}$$

Now, consider the term $\mathrm{Var}[f(x_{0})]={\hat{\sigma }}_{f}^{2}x_{0}{}^{T}{\left(X^{T}X\right)}^{-1}x_{0}$ as constant for each function at a given point. Analyzing Equation (22), we see that the variance of the estimated responses is minimized by diversification, i.e., by the uniform distribution of weights among the functions involved in the MOP. Furthermore, negative correlations between responses tend to decrease variance. We propose the following: using entropic metrics to choose the optimal weights in MOP can reduce prediction variance.

**Figure 1:**
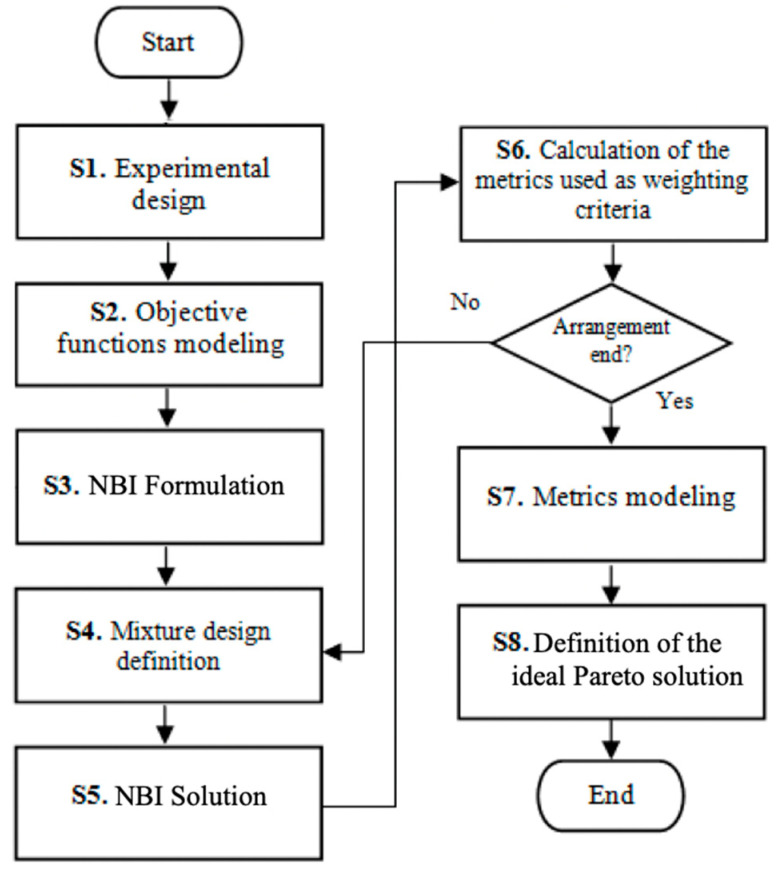
Step-by-step of the adopted methodology. Source: Adapted of Rocha et al. [55].

Figure 1 shows the step-by-step proposal.

## 4. Experimental Design 

The experimental data presented by Istadi and Amin [28] and Istadi and Amin [16] were used in this study. The authors sought to optimize a CO_2_-OCM process, by determining the condition that led to maximum CH_4_ conversion, C_2_ selectivity, and C_2_ yield.

The CaO/CeO_2_ and MnO/CeO_2_ catalysts were prepared by impregnating ceria (CeO_2_) with aqueous solutions of Ca(NO_3_)_2_ and Mn(NO_3_)_2_, respectively, as described in Istadi and Amin [56] and Istadi and Amin [16]. The catalytic performances were tested in an experimental set-up as described in Istadi and Amin [56]. The CH_4_ conversion, C_2_ selectivity, and C_2_ yield are calculated as defined by Wang et al. [26], considering that the carbon in methane is converted to C_2_H_6_, C_2_H_4_ and CO.

The most frequently used experimental design for data collection, for modeling the response surface functions, is a Central Composite Design (CCD). According to Myers et al. [36], a CCD is chosen because it is an efficient design for sequential experimentation, allowing a reasonable amount of information to test the error without requiring a large number of experiments. Additionally, the design accommodates a spherical region with five levels for each factor, which is advantageous from an experimental region point of view. The CCD was then employed in the experimental design. Using CO_2_/CH_4_ ratio (dimensionless), reactor temperature (K), wt.% CaO in ceria catalyst and wt.% MnO in ceria catalyst as the decision variables, a full factorial design 2^4^ was performed with eight axial points and two center points, generating 26 experiments. The experimental matrix is shown in Table 1 in which *x*_1_ is the CO_2_/CH_4_ ratio, *x*_2_ is the reactor temperature, *x*_3_ is the wt.% CaO in ceria catalyst, *x*_4_ is the wt.% MnO in ceria catalyst, *y*_1_ is the CH_4_ conversion (%), *y*_2_ is the C_2_ selectivity (%), and *y*_3_ is the C_2_ yield (%). The fixed experimental conditions are [16,28]: catalyst weight = 2 g; total feed flow rate = 100 mL/min; total pressure = 1 atm; gas hourly space velocity = 3000 mL/(g h).

It is important to note that the experimental matrix presented in Table 1 was planned using only 2 CP. This can be very detrimental to the stability of the prediction variance. Myers et al. [36] do not recommend using a CCD with only two CP, because this practice does not guarantee good dispersion of the prediction variance throughout the experimental region, and analyzing how the proposed method behaves in such designs is important. This was why we chose this experimental matrix.

The decision variables were analyzed in coded form. They were decoded only at the end of the analyses. This was done using the following equation [51]:

(23) $$X_{uncoded}=\frac{Hi+Lo}{2}+X_{coded}\frac{Hi-Lo}{2}$$

where: *Hi* and *Lo* are related to the values of level +1 and −1, respectively.

The parameters used in the experiments and their levels are shown in Table 2.

In the search for an optimization model that allows for the simultaneous conversion of CH_4_, C_2_ selectivity_,_ and C_2_ yield, the decision-making process, based on multiple criteria, proposed by Rocha et al. [52] can be used. The authors sought to build a uniformly distributed Pareto frontier and a way to select the preferred Pareto optimal points as the ideal solution to a given problem. The authors used the NBI method, overcoming the disadvantages of the WSM method. To verify the robustness of the final result obtained, a variance metric was adopted. Although there are different measures of prediction performance to compare experimental designs, SPV is commonly adopted [57]. However, if direct comparisons between the expected variation of estimation are desired, UPV may be more appropriate.

## 5. Results and Discussion

The analysis of experimental data shown in Table 1 generated the mathematical modeling presented in Table 3.

Table 3 shows that the *R*^2^ values indicate a good fit for the model. The lack-of-fit test is shown in Table 3. In the lack-of-fit test, small *p*-values are undesirable. At a 5% significance level, all presented models were adequate.

**Figure 2:**
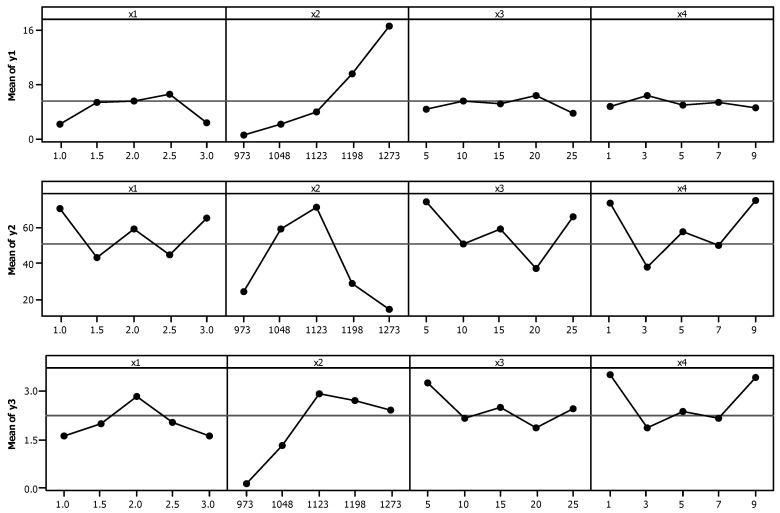
Main effect plots for CH_4_ conversion (*y*_1_), C_2_ selectivity (*y*_2_) and C_2_ yield (*y*_3_).

In order to establish a comparison of how each decision variable affects each response, the main effect plots for CH_4_ conversion (*y*_1_), C_2_ selectivity (*y*_2_) and C_2_ yield (*y*_3_) are shown in Figure 2.

According to this analysis, the reactor temperature (*x*_2_) is the most significant factor in increasing CH_4_ conversion (*y*_1_). The reactor temperature (*x*_2_) is also an important factor when analyzing C_2_ selectivity (*y*_2_) and C_2_ yield (*y*_3_), but in a different way. The reactor temperature (*x*_2_) increases the C_2_ selectivity (*y*_2_) and C_2_ yield (*y*_3_) until reaching 1123 K. When increasing the temperature above 1123 K, C_2_ selectivity (*y*_2_) and C_2_ yield (*y*_3_) decrease. The drop is greater when analyzing the values of C_2_ selectivity (*y*_2_), and this could be attributed to more CH_4_ being converted into CO rather than C_2_H_4_ and/or C_2_H_6_ [16]. For C_2_ selectivity (*y*_2_) and C_2_ yield (*y*_3_) the factors wt.% CaO in ceria catalyst (*x*_3_) and wt.% MnO in ceria catalyst (*x*_4_) showed very similar behavior, and the highest values for the responses obtained were extreme factor values, i.e., 5 and 25% CaO, and 1 and 9% MnO in ceria catalyst. The considerable impact that these factors have on C_2_ selectivity (*y*_2_) and C_2_ yield (*y*_3_) highlights the importance of the catalysts in promoting the product selectivity to C_2_H_4_ and/or C_2_H_6,_ and in inhibiting the reaction to form CO and H_2_O [16]. The CO_2_/CH_4_ ratio (*x*_1_) had different behavior for C_2_ selectivity (*y*_2_) and C_2_ yield (*y*_3_). While the highest value for C_2_ yield (*y*_3_) was observed for the CO_2_/CH_4_ ratio (*x*_1_) 2, the highest values for the C_2_ selectivity (*y*_2_) obtained were in the extreme CO_2_/CH_4_ ratio (*x*_1_) range. The 2.5 CO_2_/CH_4_ ratio (*x*_1_) led to the highest CH_4_ (*y*_1_) conversion value with a drop in the value of this response at CO_2_/CH_4_ ratio (*x*_1_) 3. The abundance of CO_2_ in the high CO_2_/CH_4_ ratio most likely decreased the catalyst activity by covering the catalyst active sites [16]. When analyzing only reactor temperature (*x*_2_), we saw that increased CH_4_ conversion (*y*_1_), C_2_ selectivity (*y*_2_) and C_2_ yield (*y*_3_) would be negatively affected, which corroborates the results presented by other authors [25,26,27]. We can see how these objectives are, thus, conflicting.

**Figure 3:**
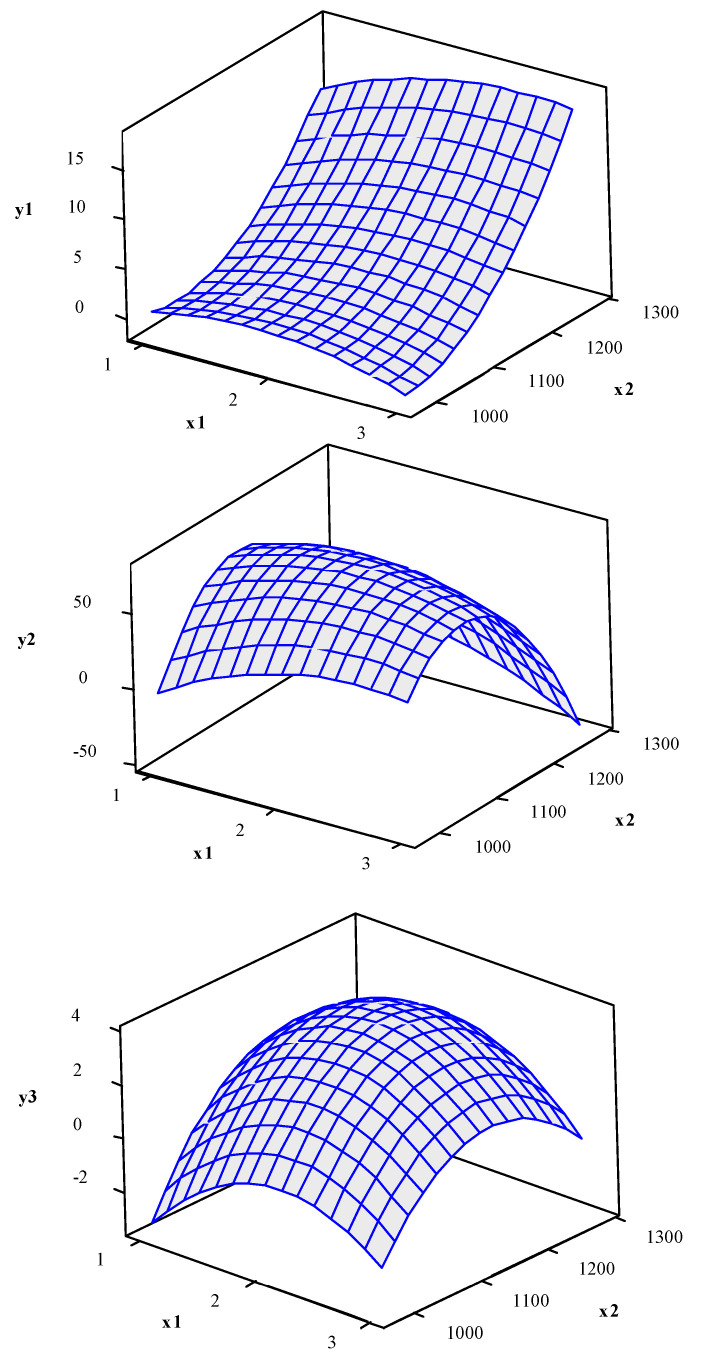
Response surface for CH_4_ conversion (*y*_1_), C_2_ selectivity (*y*_2_) and C_2_ yield (*y*_3_) (hold values: *x*_3_ = 15, *x*_4_ = 5).

Figure 3 shows the response surfaces for CH_4_ conversion (*y*_1_), C_2_ selectivity (*y*_2_) and C_2_ yield (*y*_3_).

In order to check the convexity of the functions, before performing the multi-objective optimization, the nature of the stationary point was analyzed using Equation (14). For CH_4_ conversion (*y*_1_) the eigenvalues ($\lambda_{i}$) are [1.2205; −0.5222; −0.1821; 0.1263], i.e., the different eigenvalues signs indicate that the function is neither concave nor convex and the stationary point is a saddle point. For C_2_ selectivity (*y*_2_) the eigenvalues ($\lambda_{i}$) are [−17.1859; −4.6067; −2.3090; −1.3305]. Thus, the eigenvalue signs indicate that the function is concave and the stationary point is a maximum point. For C_2_ yield (*y*_3_) the eigenvalues ($\lambda_{i}$) are [−0.7656; −0.5133; −0.2047; −0.0781]. The eigenvalue signs indicate that the function is concave and the stationary point is a maximum point. Through an analysis of the nature of the stationary point, we see that the functions have different convexities. Thus, the weighted sum method for multi-objective optimization is not adequate [58]. We therefore adopted the NBI method. To implement the NBI optimization routine, initially, the payoff matrix was estimated, obtaining the results shown in Table 4.

The payoff matrix is an underlying part of the NBI implementation. After this step, a mixture design for the weights of each objective function was defined, as presented in Table 5.

Table 5 presents the Pareto optimal set for the MOP under analysis. As shown in Rocha et al. [59], the prediction variance was affected by the weights assigned to the objectives. 

Table 6 shows the Pearson Correlation analysis between the values that were previously presented in Table 5. Using the Pearson Correlation analysis we see that the *ξ* weighting metric has a statistically negative and significant correlation with the UPV. Therefore, when maximizing this metric, the variance values tend to be lower. This information indicates that the search for the most preferred Pareto optimal point in multi-objective optimization using this metric leads to a robust response from a variability point of view.

**Figure 4:**
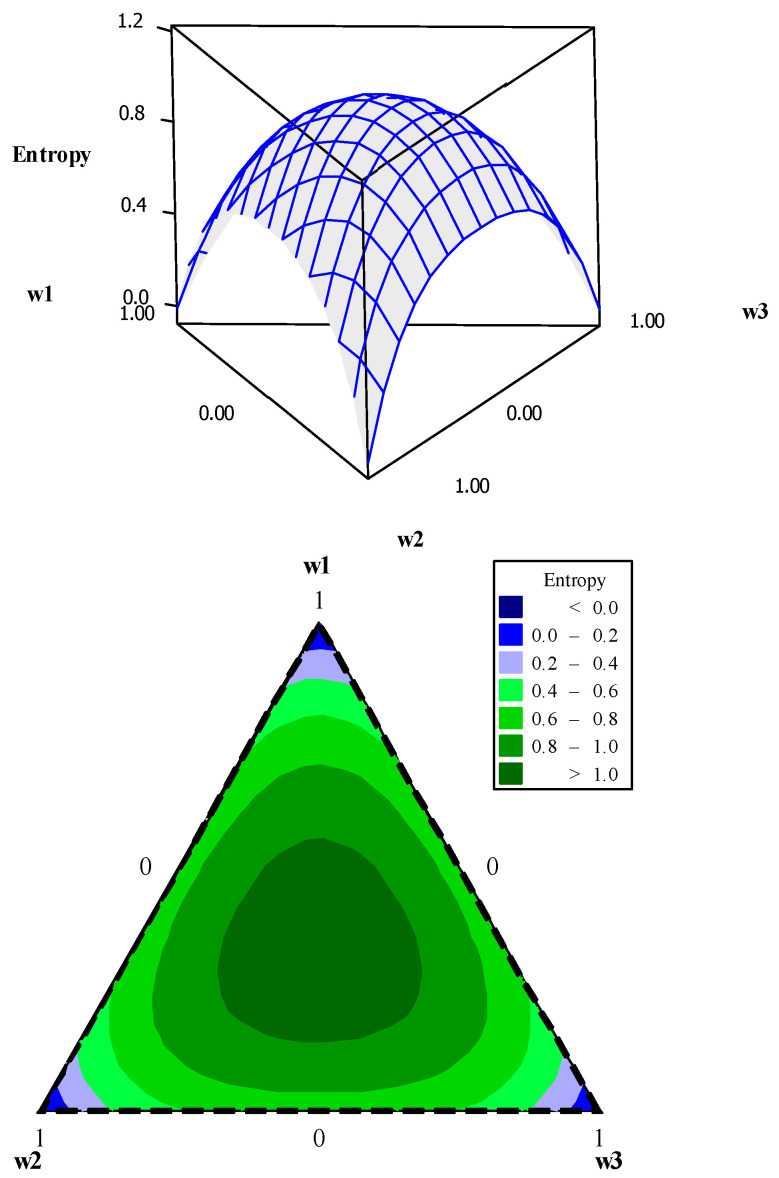
Entropy response surface and contour plot.

**Figure 5:**
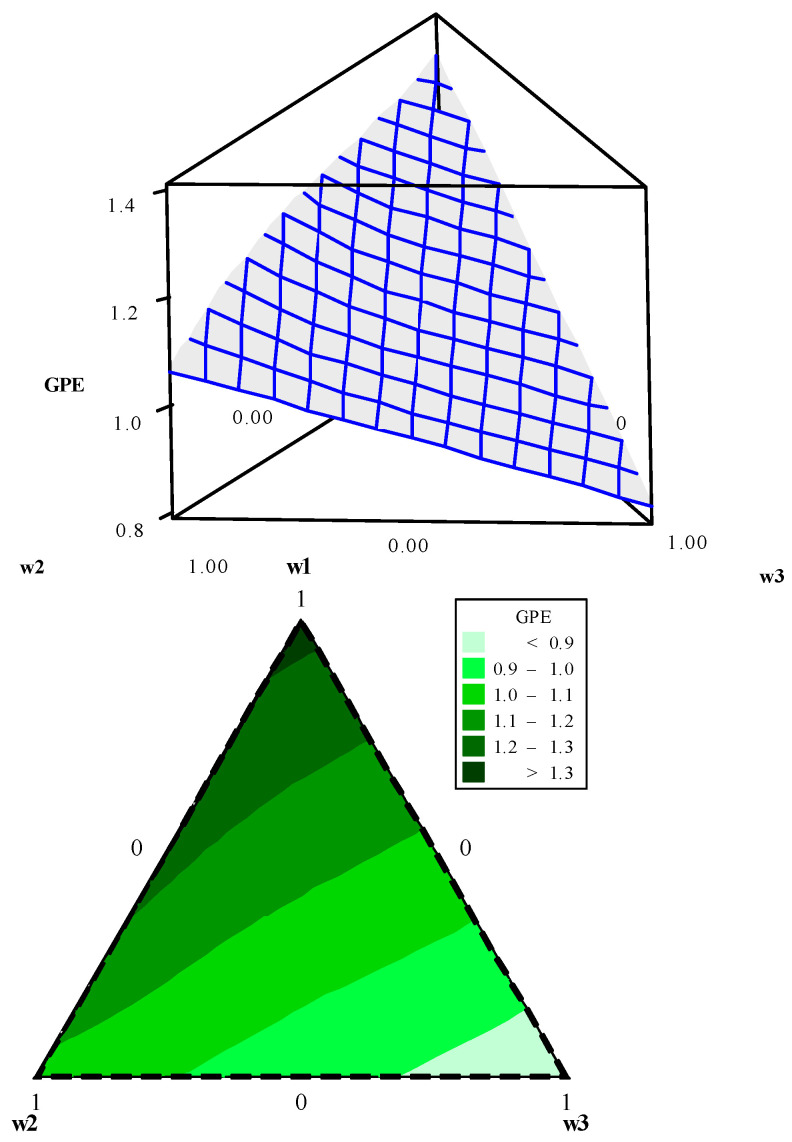
GPE response surface and contour plot.

**Figure 6:**
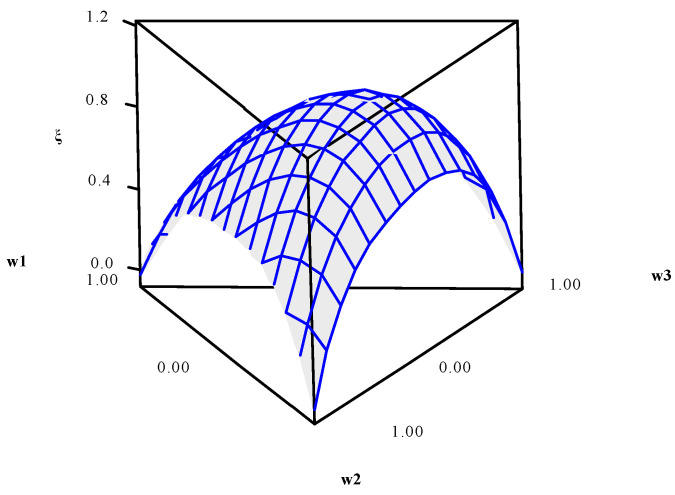

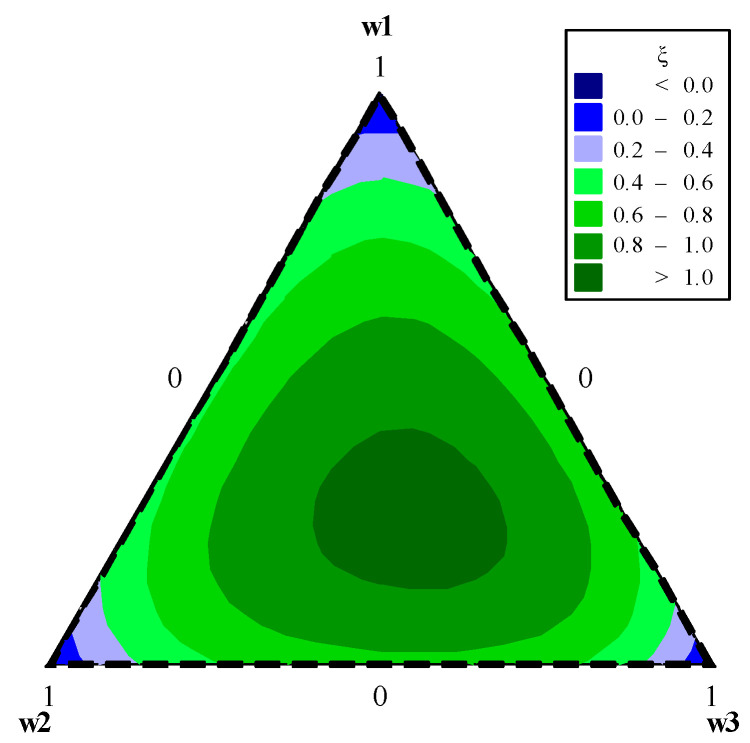
*ξ* response surface and contour plot.

**Figure 7:**
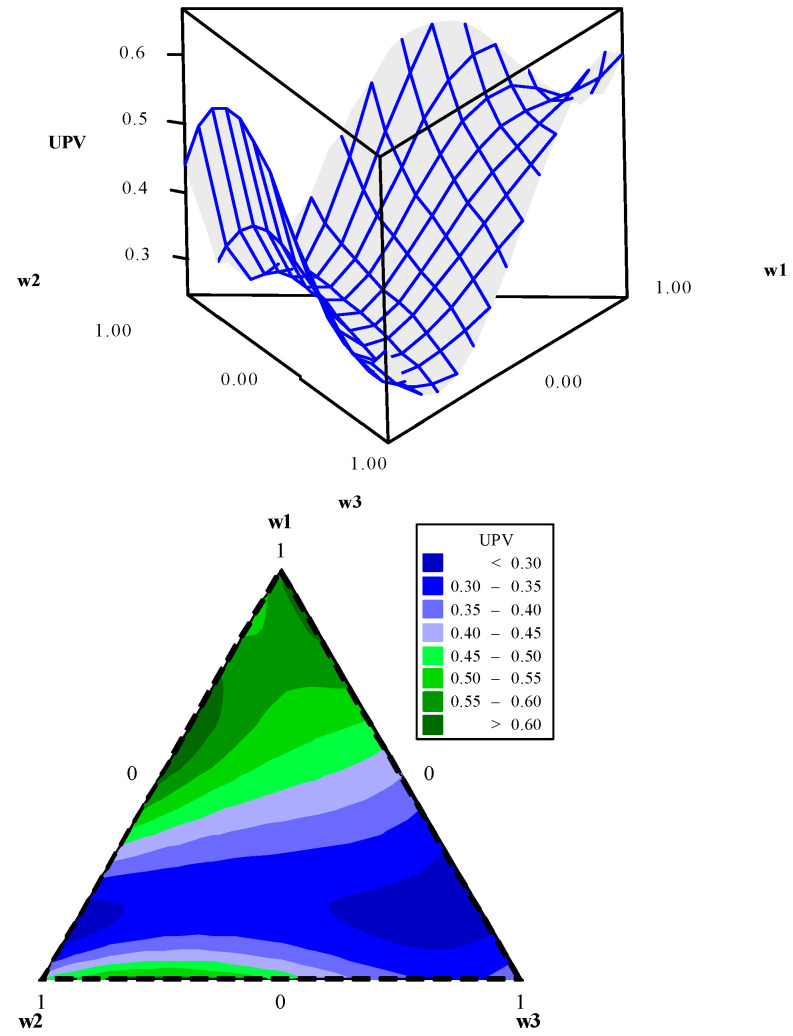
UPV response surface and contour plot.

Using data presented in Table 5, the entropy modeling, GPE, weighting metric (*ξ*), and UPV were performed. Their canonical mixing polynomials (Equations (24)–(27)), response surfaces and contour graphics were obtained (Figure 4, Figure 5, Figure 6 and Figure 7), which are presented below:

(24) $$\begin{array}{ll} Entropy= & -0.0074w_{1}-0.0074w_{2}-0.0074w_{3}+2.7705w_{1}w_{2}+2.7705w_{1}w_{3}+\\ & 2.7705w_{2}w_{3}+5.4207w_{1}w_{1}w_{2}w_{3}+5.4207w_{1}w_{2}w_{2}w_{3}+5.4207w_{1}w_{2}w_{3}w_{3}+\\ & \;1.4619w_{1}w_{2}{\left(w_{1}-w_{2}\right)}^{2}+1.4619w_{1}w_{3}{\left(w_{1}-w_{3}\right)}^{2}+1.4619w_{2}w_{3}{\left(w_{2}-w_{3}\right)}^{2} \end{array}$$

(25) $$\begin{array}{ll} GPE= & 1.3312w_{1}+1.0624w_{2}+0.8233w_{3}+0.1419w_{1}w_{2}-\\ & 0.1680w_{1}w_{2}(w_{1}-w_{2})-1.2977w_{1}w_{2}w_{2}w_{3} \end{array}$$

(26) $$\begin{array}{ll} \xi = & -0.0123w_{1}-0.0081w_{2}+0.0016w_{3}+2.2476w_{1}w_{2}+2.5539w_{1}w_{3}+2.9199w_{2}w_{3}-\\ & \;0.2272w_{1}w_{2}(w_{1}-w_{2})-0.6622w_{1}w_{3}(w_{1}-w_{3})-0.3965w_{2}w_{3}(w_{2}-w_{3})+\\ & \;3.8644w_{1}w_{1}w_{2}w_{3}+5.3402w_{1}w_{2}w_{2}w_{3}+6.2119w_{1}w_{2}w_{3}w_{3}+1.4004w_{1}w_{2}{\left(w_{1}-w_{2}\right)}^{2}+\\ & \;1.5386w_{1}w_{3}{\left(w_{1}-w_{3}\right)}^{2}+1.5177w_{2}w_{3}{\left(w_{2}-w_{3}\right)}^{2} \end{array}$$

(27) $$\begin{array}{ll} UPV= & 0.6054w_{1}+0.4386w_{2}+0.3849w_{3}+0.3919w_{1}w_{2}-0.3719w_{1}w_{3}+0.2859w_{2}w_{3}+\\ & 0.8779w_{1}w_{2}(w_{1}-w_{2})+0.9531w_{1}w_{3}(w_{1}-w_{3})+0.9960w_{2}w_{3}(w_{2}-w_{3})-\\ & 11.9662w_{1}w_{2}w_{2}w_{3}-2.6199w_{1}w_{2}{\left(w_{1}-w_{2}\right)}^{2} \end{array}$$

It is interesting to note that all canonical polynomials of mixtures showed a good fit, with *R*^2^ close to 100%. The adopted variance metric, UPV, had the worst adjustments, with an *R*^2^ value equal to 82.83%. However, this value may be acceptable [36]. This analysis highlights the fact that it is possible to model the variance metric in terms of weights. This is because the weights interfere in the solution space. However, since the optimization of distinct functions is performed simultaneously, the solution space is not the same as the initial area of the DOE, and therefore, its shape is distinguished from the Hat Matrix shape when modeling the variance.

Lastly, when maximizing *ξ*, as described in Equation (26), weights *w*_1_, *w*_2,_ and *w*_3_, related to the final solution, were identified as being *w*_1_ = 0.2603, *w*_2_ = 0.3202, and *w*_3_ = 0.4195. These optimal weights were used in a multi-objective optimization of CH_4_ conversion (*y*_1_), C_2_ selectivity (*y*_2_), and C_2_ yield (*y*_3_), reaching 8.806%, 51.468%, and 3.275%, respectively. This result is different from that obtained by Istadi and Amin [28] and Istadi and Amin [16]. When optimizing the responses using a bio-objective optimization process, the authors neglected the real trade-off behavior between the responses. The lower diversification among the responses almost led to a single-response optimization. The greatest challenge of this process is to achieve both high CH_4_ conversion (*y*_1_) and high C_2_ selectivity (*y*_2_), since it has been proven that these responses are in conflict one with the other [25,26,27]. Despite this trade-off, the results are acceptable [25] especially with respect to using low-valued natural gas.

The optimal coded values of the decision variables are CO_2_/CH_4_ ratio (*x*_1_) = 1.0060, reactor temperature (*x*_2_) = 0.7536, wt.% CaO in ceria catalyst (*x*_3_) = 0.4358, and wt.% MnO in ceria catalyst (*x*_4_) = 0.4822. Using Equation (23), the encoded values were transformed into uncoded values. Therefore, the optimal values of the decision variables are CO_2_/CH_4_ ratio (*x*_1_) = 2.50, reactor temperature (*x*_2_) = 1179.5 K, wt.% CaO in ceria catalyst (*x*_3_) = 17.2%, and wt.% MnO in ceria catalyst (*x*_4_) = 6.0%. The high CO_2_/CH_4_ ratio (*x*_1_) favors CH_4_ conversion (*y*_1_) [26]. A high reactor temperature (*x*_2_) leads to high CH_4_ conversion (*y*_1_), but the C_2_ yield (*y*_3_) decreases at reactor temperatures higher than 1173 K, due to low C_2_ selectivity (*y*_2_) [26,27]. The high wt.% CaO (*x*_3_) and wt.% MnO (*x*_4_) in ceria catalyst increases CH_4_ conversion (*y*_1_) and C_2_ selectivity (*y*_2_). According to Wang and Ohtsuka [27], these catalysts can activate CO_2_ to produce active oxygen for CH_4_ conversion (*y*_1_) and their basicity leads to improvements in C_2_ selectivity (*y*_2_).

**Figure 8:**
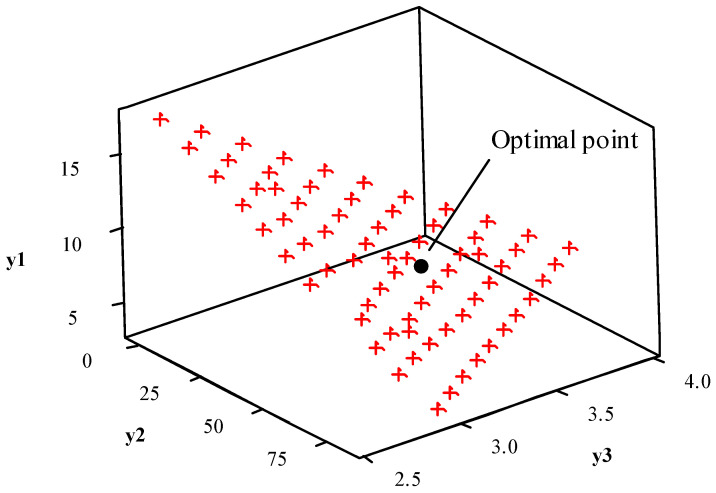
Pareto frontier for CH_4_ conversion (*y*_1_), C_2_ selectivity (*y*_2_) and C_2_ yield (*y*_3_) optimization.

The data presented in Table 5 were used to construct Figure 8. Here, the Pareto frontier constructed using the NBI method is presented, with the ideal point highlighted.

Figure 8 shows the uniform distribution of the optimal Pareto points on the frontier. This also presents the most preferrable Pareto point, which is the final solution for the MOP. The confidence interval values are shown in Table 7. 

For α=5%, the confidence interval of 100(1−α)%, for the mean response, at point $x_{0}{}^{T}=\left[\begin{array}{ccccc} 1 & x_{01} & x_{02} & \ldots & x_{0k} \end{array}\right]$ is:

(28) $$\hat{y}(x_{0})-t_{\alpha /2,\;n-p}\sqrt{{\hat{\sigma }}^{2}x_{0}{}^{T}{\left(X^{T}X\right)}^{-1}x_{0}}\leq \mu_{y|x_{0}}\leq \hat{y}(x_{0})+t_{\alpha /2,\;n-p}\sqrt{{\hat{\sigma }}^{2}x_{0}{}^{T}{\left(X^{T}X\right)}^{-1}x_{0}}$$

When analyzing the question involving variability, the final solution obtained that maximizes the *ξ* metric is robust, since this metric leads the solution in a region of minimum variance, with less variability, and greater reliability. However, the confidence interval of the C_2_ selectivity (*y*_2_) is higher than the other responses. This demonstrates that C_2_ selectivity (*y*_2_) is the most difficult parameter to control in the process.

**Figure 9:**
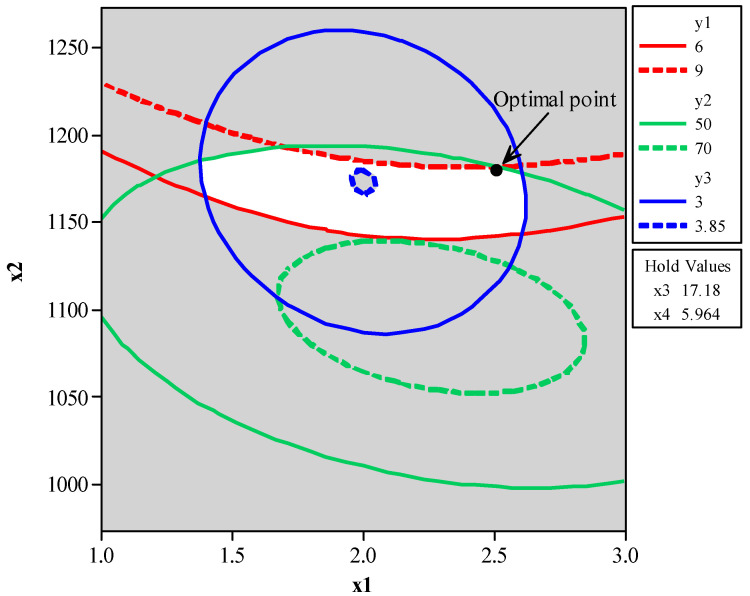
Point of optimization for CH_4_ conversion (*y*_1_), C_2_ selectivity (*y*_2_), and C_2_ yield (*y*_3_) optimization.

Figure 9 shows the overlap of the different objective functions defining the feasible region for the analyzed problem.

Figure 9 shows the conflicting nature between CH_4_ conversion (*y*_1_) and C_2_ selectivity (*y*_2_). An increase in CH_4_ conversion (*y*_1_) leads to a decrease in C_2_ selectivity (*y*_2_). In this study, the optimum point was chosen based on the maximization of metric *ξ*, and still, it was shown to be the most robust point. However, one can see that the region of maximum C_2_ yield (*y*_3_) lies within the feasible region for the problem, and is obtainable, even though this is not the most reliable, from a statistical process control point of view.

## 6. Conclusions

This paper studied direct low-value natural gas conversion using carbon dioxide oxidative coupling of methane over CaO/CeO_2_ and MnO/CeO_2_ catalysts to produce C_2_ hydrocarbons. The NBI method was adopted to simultaneously optimize CH_4_ conversion, C_2_ selectivity, and C_2_ yield. This method helped build an evenly distributed Pareto frontier for the three responses, regardless of the convexity of the functions.

The mathematical response model showed acceptable fitting, and the functions were proven adequate. Some results described in literature have been corroborated, e.g., the positive influence of reactor temperature in CH_4_ conversion, the negative influence of reactor temperature in C_2_ selectivity, the synergistic effect between reactor temperature, the wt.% CaO in ceria catalyst in increasing CH_4_ conversion and C_2_ yield, and the synergistic effect between reactor temperature and wt.% MnO in ceria catalyst in increasing C_2_ yield.

An entropic measure was used to select the most preferred ideal Pareto point for the final solution. The decision-making criteria was useful and fundamental in identifying and mapping regions with minimum variation within the optimal Pareto responses obtained in the optimization processes. Furthermore, this study demonstrates that the weights used in the multi-objective optimization process have an influence on the variation in the forecast of the responses obtained. This study also proves the robustness of the weighting process used in choosing the final solution.

The simultaneous optimal values for the objective functions were CH_4_ conversion = 8.806%, C_2_ selectivity = 51.468%, and C_2_ yield = 3.275%. These results were obtained by using the following process parameter combinations: CO_2_/CH_4_ ratio = 2.50, reactor temperature = 1179.5 K, wt.% CaO in ceria catalyst = 17.2%, and wt.% MnO in ceria catalyst = 6.0%. The analyses of the confidence interval for the responses showed that C_2_ selectivity has greater variability, and was the most difficult parameter to control in the process.

From an environmental point of view, this is an efficient process since it helps reduce CO_2_ and CH_4_ emissions and reduces waste, as hydrocarbon resources, specifically from low-value natural gas, can be used.

## Figures and Tables

**Table 1:** CCD for CH_4_ conversion (*y*_1_), C_2_ selectivity (*y*_2_) and C_2_ yield (*y*_3_).

N	*x*_1_	*x*_2_	*x*_3_	*x*_4_	*y*_1_	*y*_2_	*y*_3_
1	1.5	1048	10	3	2.63	69.01	1.82
2	2.5	1048	10	3	2.68	60.20	1.61
3	1.5	1198	10	3	7.95	32.71	2.60
4	2.5	1198	10	3	9.74	18.73	1.82
5	1.5	1048	20	3	1.76	24.62	0.43
6	2.5	1048	20	3	2.92	55.95	1.63
7	1.5	1198	20	3	9.92	27.84	2.76
8	2.5	1198	20	3	13.41	16.21	2.17
9	1.5	1048	10	7	2.20	78.15	1.72
10	2.5	1048	10	7	2.29	78.37	1.80
11	1.5	1198	10	7	7.80	35.98	2.81
12	2.5	1198	10	7	8.70	33.12	2.88
13	1.5	1048	20	7	1.25	42.64	0.53
14	2.5	1048	20	7	1.55	64.79	1.00
15	1.5	1198	20	7	9.03	34.62	3.13
16	2.5	1198	20	7	10.89	30.78	3.35
17	1.0	1123	15	5	2.27	70.51	1.60
18	3.0	1123	15	5	2.47	65.18	1.61
19	2.0	973	15	5	0.54	24.30	0.13
20	2.0	1273	15	5	16.59	14.32	2.38
21	2.0	1123	5	5	4.33	74.63	3.23
22	2.0	1123	25	5	3.70	66.30	2.45
23	2.0	1123	15	1	4.71	74.07	3.49
24	2.0	1123	15	9	4.53	75.24	3.41
25	2.0	1123	15	5	4.81	72.58	3.49
26	2.0	1123	15	5	5.06	75.64	3.83

**Table 2:** Parameters used in the experiments.

Factors	Symbol	Levels				
		−2	−1	0	1	2
CO_2_/CH_4_ ratio	*x*_1_	1	1.5	2	2.5	3
Reactor temperature (K)	*x*_2_	973	1048	1123	1198	1273
wt.% CaO (%)	*x*_3_	5	10	15	20	25
wt.% MnO (%)	*x*_4_	1	3	5	7	9

**Table 3:** Objective functions mathematical models.

Terms	*y*_1_	*y*_2_	*y*_3_
Constant	**4.9350**	**74.1100**	**3.6600**
*x*_1_	0.4183	0.0800	**0.0200**
*x*_2_	**3.8442**	**−10.9875**	0.6450
*x*_3_	0.2283	−5.2283	−0.1508
*x*_4_	−0.3192	3.9800	0.0925
*x*_1_ *x*_1_	−0.4700	−3.9140	**−0.5610**
*x*_2_ *x*_2_	**1.0788**	**−16.0477**	**−0.6485**
*x*_3_ *x*_3_	−0.0588	−3.2590	**−0.2523**
*x*_4_ *x*_4_	0.0925	−2.2115	−0.0998
*x*_1_ *x*_2_	0.4025	−4.8250	−0.1638
*x*_1_ *x*_3_	0.2488	3.9650	0.1338
*x*_1_ *x*_4_	−0.2088	1.1725	0.0763
*x*_2_ *x*_3_	**0.7113**	5.4150	**0.2913**
*x*_2_ *x*_4_	−0.1188	−0.9475	**0.2038**
*x*_3_ *x*_4_	−0.2050	0.2025	−0.0212
*p*-value	0.000	0.030	0.000
*R*^2^ (%)	97.47%	80.29%	94.82%
Lack-of-fit	0.131	0.106	0.483

The values presented in bold represent the significant terms of the model (thus, *p*-value < 5%).

**Table 4:** Payoff matrix for the objective functions.

*y*_1_	*y*_2_	*y*_3_
**17.118**	0.000	2.619
3.376	**82.614**	2.945
7.793	59.630	**3.933**

Bold values represent individual optimums.

**Table 5:** Mixture design.

Weights			*y*_1_	*y*_2_	*y*_3_	*Entropy*	*GPE*	ξ	*UPV*
*w*_1_	*w*_2_	*w*_3_							
1.000	0.000	0.000	17.118	0.000	2.619	0.0000	1.3341	0.0000	0.5833
0.900	0.100	0.000	15.744	8.261	2.652	0.3251	1.3061	0.2489	0.5833
0.900	0.000	0.100	16.185	5.963	2.750	0.3251	1.2830	0.2534	0.5833
0.800	0.200	0.000	14.370	16.523	2.684	0.5004	1.2781	0.3915	0.5833
0.800	0.100	0.100	14.811	14.224	2.783	0.6390	1.2550	0.5092	0.5833
0.800	0.000	0.200	15.253	11.926	2.882	0.5004	1.2319	0.4062	0.5833
0.700	0.300	0.000	12.995	24.784	2.717	0.6109	1.2501	0.4887	0.5833
0.700	0.200	0.100	13.437	22.486	2.816	0.8018	1.2270	0.6535	0.5833
0.700	0.100	0.200	13.879	20.187	2.914	0.8018	1.2039	0.6660	0.5833
0.700	0.000	0.300	14.320	17.889	3.013	0.6109	1.1807	0.5173	0.5833
0.600	0.400	0.000	11.621	29.734	2.749	0.6730	1.2622	0.5332	0.5833
0.600	0.300	0.100	12.063	30.747	2.848	0.8979	1.1990	0.7489	0.5833
0.600	0.200	0.200	12.505	28.449	2.947	0.9503	1.1758	0.8082	0.5833
0.600	0.100	0.300	12.946	26.151	3.046	0.8979	1.1527	0.7790	0.5833
0.600	0.000	0.400	13.388	23.852	3.145	0.6730	1.1296	0.5958	0.5833
0.500	0.500	0.000	10.247	36.364	2.782	0.6931	1.2539	0.5528	0.5833
0.500	0.400	0.100	10.689	36.248	2.881	0.9433	1.2044	0.7833	0.5833
0.500	0.300	0.200	11.131	36.710	2.980	1.0297	1.1478	0.8970	0.5569
0.500	0.200	0.300	11.572	34.412	3.078	1.0297	1.1247	0.9155	0.3936
0.500	0.100	0.400	12.014	32.113	3.177	0.9433	1.1016	0.8563	0.3420
0.500	0.000	0.500	12.456	29.815	3.276	0.6931	1.0785	0.6427	0.3215
0.400	0.600	0.000	8.873	42.931	2.815	0.6730	1.2464	0.5400	0.5833
0.400	0.500	0.100	9.315	42.340	2.913	0.9433	1.2026	0.7844	0.5833
0.400	0.400	0.200	9.756	44.972	3.012	1.0549	1.1198	0.9420	0.5118
0.400	0.300	0.300	10.198	42.673	3.111	1.0889	1.0967	0.9929	0.4537
0.400	0.200	0.400	10.640	40.375	3.210	1.0549	1.0736	0.9826	0.3384
0.400	0.100	0.500	11.082	38.077	3.309	0.9433	1.0505	0.8980	0.3119
0.400	0.000	0.600	11.523	35.778	3.407	0.6730	1.0274	0.6551	0.3117
0.300	0.700	0.000	7.499	57.830	2.891	0.6109	1.1269	0.5421	0.3459
0.300	0.600	0.100	7.941	55.531	2.946	0.8979	1.1149	0.8054	0.3320
0.300	0.500	0.200	8.382	53.233	3.045	1.0297	1.0918	0.9431	0.3223
0.300	0.400	0.300	8.824	50.935	3.144	1.0889	1.0687	1.0189	0.3171
0.300	0.300	0.400	9.266	48.636	3.242	1.0889	1.0456	1.0414	0.3142
0.300	0.200	0.500	9.707	46.338	3.341	1.0297	1.0225	1.0070	0.3126
0.300	0.100	0.600	10.149	44.040	3.440	0.8979	0.9994	0.8985	0.3120
0.300	0.000	0.700	10.591	41.741	3.539	0.6109	0.9763	0.6257	0.3115
0.200	0.800	0.000	6.125	63.589	2.880	0.5004	1.1403	0.4388	0.3226
0.200	0.700	0.100	6.566	62.391	2.978	0.8018	1.1039	0.7264	0.3162
0.200	0.600	0.200	6.927	61.494	3.077	0.9503	1.0686	0.8893	0.3119
0.200	0.500	0.300	7.450	59.196	3.176	1.0297	1.0407	0.9894	0.3107
0.200	0.400	0.400	7.892	56.898	3.275	1.0549	1.0176	1.0367	0.3108
0.200	0.300	0.500	8.333	54.599	3.374	1.0297	0.9945	1.0354	0.3114
0.200	0.200	0.600	8.775	52.301	3.473	0.9503	0.9714	0.9783	0.3120
0.200	0.100	0.700	9.217	50.003	3.571	0.8018	0.9483	0.8456	0.3122
0.200	0.000	0.800	9.658	47.704	3.670	0.5004	0.9252	0.5409	0.3124
0.100	0.900	0.000	4.751	69.736	2.912	0.3251	1.1379	0.2857	0.3116
0.100	0.800	0.100	5.192	68.568	3.011	0.6390	1.1011	0.5803	0.3107
0.100	0.700	0.200	5.634	67.371	3.110	0.8018	1.0647	0.7531	0.3132
0.100	0.600	0.300	6.076	66.135	3.209	0.8979	1.0287	0.8729	0.3185
0.100	0.500	0.400	6.448	65.159	3.308	0.9433	0.9936	0.9494	0.3271
0.100	0.400	0.500	6.959	62.861	3.406	0.9433	0.9665	0.9761	0.3275
0.100	0.300	0.600	7.401	60.562	3.505	0.8979	0.9434	0.9519	0.3280
0.100	0.200	0.700	7.843	58.264	3.604	0.8018	0.9203	0.8713	0.3274
0.100	0.100	0.800	8.284	55.966	3.703	0.6390	0.8971	0.7123	0.3268
0.100	0.000	0.900	8.726	53.667	3.802	0.3251	0.8740	0.3719	0.3237
0.000	1.000	0.000	3.377	82.614	2.945	0.0000	1.0540	0.0000	0.3984
0.000	0.900	0.100	3.818	80.315	3.044	0.3251	1.0309	0.3153	0.5597
0.000	0.800	0.200	4.260	78.017	3.142	0.5004	1.0078	0.4965	0.5833
0.000	0.700	0.300	4.702	75.719	3.241	0.6109	0.9847	0.6203	0.5833
0.000	0.600	0.400	5.097	73.420	3.340	0.6730	0.9643	0.6979	0.5833
0.000	0.500	0.500	5.447	71.122	3.439	0.6931	0.9465	0.7323	0.5122
0.000	0.400	0.600	5.834	68.824	3.538	0.6730	0.9266	0.7263	0.3236
0.000	0.300	0.700	6.319	66.525	3.637	0.6109	0.9010	0.6780	0.3541
0.000	0.200	0.800	6.910	64.227	3.735	0.5004	0.8691	0.5757	0.3964
0.000	0.100	0.900	7.352	61.929	3.834	0.3251	0.8460	0.3842	0.3562
0.000	0.000	1.000	7.794	59.631	3.933	0.0000	0.8229	0.0000	0.3106
0.333	0.333	0.333	9.429	47.415	3.166	1.0986	1.0703	1.0264	0.3107
0.667	0.167	0.167	13.274	23.707	2.892	0.8676	1.2022	0.7216	0.3697
0.167	0.667	0.167	6.403	63.621	3.055	0.8676	1.0790	0.8040	0.3109
0.167	0.167	0.667	8.611	53.523	3.549	0.8676	0.9466	0.9165	0.3131

**Table 6:** Pearson correlation coefficients.

	*Entropy*	*GPE*	ξ
***GPE***	−0.008		
	0.950		
ξ	0.967	−0.232	
	0.000	0.054	
***UPV***	−0.228	0.642	−0.378
	0.057	0.000	0.001

**Table 7:** Confidence interval values for the responses.

Responses	Lower Limit	Mean	Upper Limit
*y*_1_	7.583	8.806	10.029
*y*_2_	32.802	51.468	70.133
*y*_3_	2.845	3.275	3.704

## Data Availability

Not applicable.
